# Visualization of protein crystals by high-energy phase-contrast X-ray imaging

**DOI:** 10.1107/S2059798319011379

**Published:** 2019-10-31

**Authors:** Maxim Polikarpov, Gleb Bourenkov, Irina Snigireva, Anatoly Snigirev, Sophie Zimmermann, Krisztian Csanko, Sandor Brockhauser, Thomas R. Schneider

**Affiliations:** a European Molecular Biology Laboratory, Hamburg Unit c/o DESY, Building 25A, Notkestrasse 85, 22607 Hamburg, Germany; b European Synchrotron Radiation Facility, 71 Avenue des Martyrs, 38043 Grenoble, France; c X-ray Optics and Physical Materials Science Laboratory, Immanuel Kant Baltic Federal University, Nevskogo 14, Kaliningrad 236041, Russian Federation; d BASF SE, Pfalzgrafenstrasse 1, 67061 Ludwigshafen, Germany; e Biological Research Centre (BRC), Hungarian Academy of Sciences, Temesvári krt. 62, Szeged 6726, Hungary; f European XFEL, Holzkoppel 4, 22869 Schenefeld, Germany

**Keywords:** phase-contrast X-ray imaging, lipidic cubic phase, X-ray tomography, X-ray refractive lenses

## Abstract

For the extraction of the best possible X-ray diffraction data from macromolecular crystals, accurate positioning of the crystals with respect to the X-ray beam is crucial. In addition, information about the shape and internal defects of crystals allows the optimization of data-collection strategies. Here, it is demonstrated that the X-ray beam available on the macromolecular crystallo­graphy beamline P14 at the high-brilliance synchrotron-radiation source PETRA III at DESY, Hamburg, Germany can be used for high-energy phase-contrast microtomography of protein crystals mounted in an optically opaque lipidic cubic phase matrix. Three-dimensional tomograms have been obtained at X-ray doses that are substantially smaller and on time scales that are substantially shorter than those used for diffraction-scanning approaches that display protein crystals at micrometre resolution. Adding a compound refractive lens as an objective to the imaging setup, two-dimensional imaging at sub-micrometre resolution has been achieved. All experiments were performed on a standard macromolecular crystallography beamline and are compatible with standard diffraction data-collection workflows and apparatus. Phase-contrast X-ray imaging of macromolecular crystals could find wide application at existing and upcoming low-emittance synchrotron-radiation sources.

## Abstract 2

High-energy phase-contrast X-ray microscopy and tomography of protein crystals in an optically opaque matrix is demonstrated with micrometre resolution on the macromolecular crystallography beamline P14 at PETRA III.

## 1. Introduction   

A crucial step in setting up a successful X-ray diffraction experiment is the accurate centering of the crystal of interest with respect to the X-ray beam. For cases where crystals are optically visible, high-resolution optical microscopy in combination with highly accurate diffractometer mechanics and easy-to-use user interfaces allow users to conveniently and accurately center crystals, as for example implemented in the on-axis viewing system used in combination with the MD2/3 diffractometers (Cipriani *et al.*, 2007 ▸). However, for macromolecular crystals embedded in a buffer solution, reflection and/or refraction by the material surrounding the crystals can render the crystals invisible and/or indicate an incorrect spatial position (Bowler *et al.*, 2016 ▸; Axford *et al.*, 2012 ▸; Wagner *et al.*, 2009 ▸).

For the centering of optically invisible, often small (<20 µm in linear dimensions) crystals, many synchrotron beamlines have implemented automatic procedures based on rastering the sample for diffraction with a small X-ray beam (Cherezov *et al.*, 2009 ▸; Bowler *et al.*, 2010 ▸; Aishima *et al.*, 2010 ▸; Hilgart *et al.*, 2011 ▸; Hirata *et al.*, 2013 ▸). These procedures have proven to be particularly useful for diffraction data collection from crystals grown and mounted in lipidic cubic phase (LCP; Landau & Rosenbusch, 1996 ▸; Caffrey, 2000 ▸) or *in meso* phase (Caffrey, 2003 ▸) and subsequently cryocooled. For such mounts, the matrix surrounding the crystals becomes opaque, making the optical localization of crystals impossible (Cherezov *et al.*, 2009 ▸). While crystals can be localized using X-ray rastering schemes, there are several drawbacks to this method. Firstly, a significant portion of the tolerable X-ray dose is used just to localize the crystals; secondly, to localize the sample in three dimensions at least two raster scans have to be performed at different orientations of the sample mount, further increasing the X-ray dose spent on localization; and thirdly, the positional resolution of raster scans is inherently limited by the dimensions of the X-ray beam (typically 5–10 µm), while micrometre-sized crystals would need to be located with micrometre accuracy. For very small crystals, the entire tolerable dose may be needed to acquire a single interpretable diffraction pattern, implying the use of serial crystallography approaches (Gati *et al.*, 2014 ▸).

Second-order nonlinear optical imaging of chiral crystals (SONICC; Wampler *et al.*, 2008 ▸; Kissick *et al.*, 2011 ▸) has been used successfully for the detection of integral membrane-protein crystals in lipidic mesophases (Kissick *et al.*, 2010 ▸) and for the localization of crystals on a diffractometer (Kissick *et al.*, 2013 ▸). However, this approach requires a dedicated additional laser illumination and detection system to be installed in a potentially already crowded microfocus diffractometer environment.

In 2013, Warren and coworkers addressed the visualization of macromolecular crystals in lipidic cubic phase using X-ray microradiography and microtomography (Warren *et al.*, 2013 ▸). From their study, it was concluded that significant absorption contrast could be observed for crystals with a thickness down to 5 µm using a radiation dose smaller but comparable to the dose required for a single grid-scan.

Given the significant fraction of coherent X-rays in high-energy (>10 keV) radiation produced by high-brilliance synchrotrons such as PETRA III (Franz *et al.*, 2006 ▸; Balewski *et al.*, 2004 ▸), the use of phase contrast to image macromolecular crystals in a surrounding matrix could be attempted. Full-field phase-contrast X-ray imaging can be considered as in-line holography (Snigirev *et al.*, 1995 ▸), where a coherent reference wave interferes with the waves influenced by the sample. The interference gives rise to an image that highlights interfaces between regions of different electron density inside the sample, whereby for soft-matter objects higher energy X-rays are more effective in terms of increased penetration depth and reduced absorbed dose (Als-Nielsen & McMorrow, 2011 ▸). In dedicated imaging experiments, phase-contrast full-field imaging in the hard X-ray region has been successfully used in imaging soft-matter objects at high resolution in tomographic settings (Salditt *et al.*, 2017 ▸; Töpperwien *et al.*, 2018 ▸), even enabling time-resolved three-dimensional observations of biological processes (Moosmann *et al.*, 2013 ▸; Walker *et al.*, 2014 ▸).

For full-field phase-contrast X-ray imaging, two essential requirements are (i) a small radiation source providing high transversal coherence and (ii) homogeneous illumination of the sample (Nugent *et al.*, 2008 ▸; Arhatari *et al.*, 2009 ▸). Concerning the relation between X-ray source size and the level of transversal coherence, denoting λ as the wavelength of the radiation, *d* as the size of the X-ray source [full width at half maximum (FWHM) of its intensity profile] and *L* as the distance from the source to the observer, the transverse coherence length *l*_c_ can be estimated as
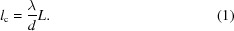



The coherence length should be considered in relation to the resolution limit, Δ, that one plans to achieve in phase-contrast X-ray imaging. Nugent *et al.* (2008 ▸) have shown that phase-contrast imaging has essentially coherent behavior when *l*_c_ is larger than Δ by approximately a factor of 15. To reach a resolution of Δ = 1 µm on a beamline operating at λ ≃ 1 Å with a source–sample distance of *L* = 60 m, the source size should therefore be less than 400 µm. The second requirement, homogeneous illumination of the sample, has been difficult to meet in the past on many beamlines as high heat loads on monochromator crystals and the use of reflective X-ray optics have resulted in beam profiles with significant structure and instabilities. With respect to phase-contrast imaging, imperfections in the optical components in a beamline reduce the transversal coherence length.

In 2008, Brockhauser and coworkers reported a set of successful X-ray microtomography experiments (Brock­hauser *et al.*, 2008 ▸) on macromolecular crystals performed at the European Synchrotron Radiation Facility on the imaging beamline ID15A (Michiel *et al.*, 2005 ▸) at 55 keV and on the macromolecular crystallography beamline ID14-4 (McCarthy *et al.*, 2009 ▸) at 12.7 keV. With the goal of providing three-dimensional crystal shapes for analytical X-ray absorption corrections for use in crystallographic data processing, they succeeded in determining the three-dimensional shapes of macromolecular crystals, including the surrounding matrix of vitrified solution and the sample holder, by a combination of absorption and phase contrast with data collected at multiple sample-to-detector distances. While the dimensions of the imaged crystals were 40–210 µm, allowing straightforward visualization, the resolution of the tomograms obtained was sufficient to detect cracks inside the crystals on a scale of 2 µm. Although these experiments showed the principal feasibility of using X-ray imaging to visualize macromolecular crystals, technical challenges in terms of the level of coherence at 12.7 keV, inhomogeneous beam profiles and beam-intensity fluctuations precluded practical use at the time.

**Figure 1:**
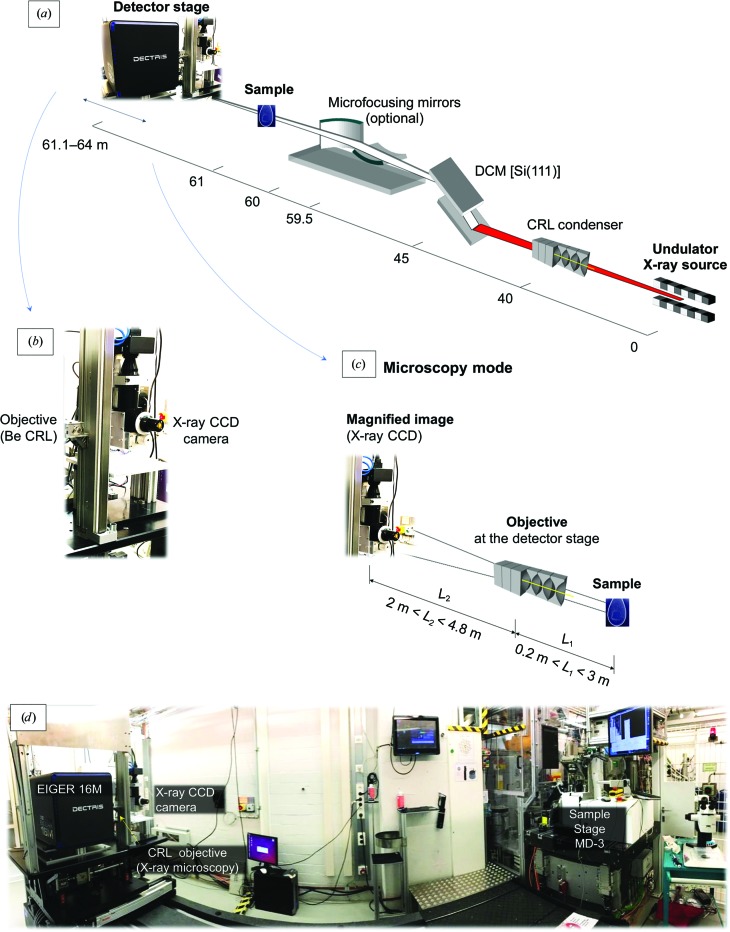
Beamline P14 at EMBL Hamburg. (*a*) The first optical element, a transfocator, is positioned 40 m from the source point. The double-crystal monochromator is located at a distance of 45 m from the source. The X-ray beam can be additionally focused at the sample position (61 m from the source) using bimorph X-ray mirrors in KB configuration located 60 m from the source. The detector stage carries detectors for crystallography and X-ray imaging, whereby the available motorized degrees of freedom can be used to choose between the two detector systems. (*b*) As a third positional option, refractive X-ray lenses are also mounted on the detector stage which can be used to support magnified X-ray imaging. (*c*) For magnified X-ray imaging, the detector can be mounted on the downstream hutch wall while refractive X-ray lenses are positioned inline. (*d*) Overview of the experimental hutch.

With the current generation of high-brilliance synchrotrons and beamlines, many of the previous limitations can be overcome. For example, on the EMBL beamline P14 at PETRA III at DESY, Hamburg, Germany, a U29 undulator (Barthelmess *et al.*, 2008 ▸) at an X-ray energy of 12 keV nominally provides an X-ray beam with source dimensions of 13 × 330 µm, a divergence of 10 × 20 µrad (vertical × horizontal, FWHM) and a total flux of 2 × 10^13^ photons s^−1^ [with a Si(111) monochromator]. At the standard sample position on beamline P14, at a distance of 61 m from the source point (Fig. 1 ▸), these beam characteristics allow the full photon flux to be delivered into a cross-section of 0.6 × 1.2 mm (FWHM). The expected transversal coherence lengths at an energy of 12 keV at the sample position are of the order of 500 and 20 µm along the vertical and the horizontal directions, respectively, while the central 500 × 500 µm region of the beam provides quasi-homogeneous illumination conditions. In the context of crystallographic data collection, this beam has been used for high-quality data collection from ‘large’ (>100 µm) macromolecular crystals (Santos *et al.*, 2012 ▸).

In the following, we demonstrate that the X-ray beam available on the macromolecular crystallography beamline P14 at PETRA III at DESY, Hamburg, Germany is of sufficient quality for imaging protein crystals at micrometre resolution with requirements in dose and wall-clock time that are compatible with crystallographic data collection from the same sample.

## 2. Materials and methods   

### 2.1. Protein crystals   

Hen egg-white lysozyme (Sigma) was dissolved in Milli-Q water to yield a protein solution at 50 mg ml^−1^. The mesophase was produced by melting monoacylglycerol (MAG) lipid monoolein (9.9 MAG; Jena Bioscience) at ∼318 K and subsequently homogenizing one volume of lysozyme solution with 1.5 volumes of the monoolein in a coupled-syringe mixing device (Aherne *et al.*, 2012 ▸). The protein-loaded mesophase was dispensed robotically into the wells of a Laminex UV Plastic Base 100 Micron crystallization plate (Molecular Dimensions) at 293 K using 50 nl mesophase and 800 nl precipitant solution with a Mosquito LCP robot (TTP Labtech) and sealed using a Laminex UV Plastic 200 µm Film Cover. The precipitant solution consisted of 100 m*M* sodium acetate pH 4.5, 15–26%(*v*/*v*) PEG 400, 0.5–1 *M* NaCl. Crystals grew to maximum linear dimensions of 50 µm within 24 h at 293 K.

### 2.2. Experimental setup   

All experiments were carried out on the EMBL beamline P14 at the PETRA III storage ring, DESY, Hamburg, Germany. P14 uses a standard U29 undulator (Barthelmess *et al.*, 2008 ▸) with a nominal source size of approximately 13 × 330 µm (FWHM) in the vertical and horizontal directions, respectively. Employing a liquid-nitrogen-cooled vertical offset double-crystal Si(111) monochromator, P14 can operate with X-ray energies ranging from 6 to 30 keV. The beamline layout is shown in Fig. 1 ▸.

On beamline P14, the size, shape and intensity of the X-ray beam can be adjusted by using refractive and/or reflective optical elements. For standard crystallographic applications, requiring ‘large’ beam sizes ranging from 20 to 200 µm, a white-beam transfocator (Vaughan *et al.*, 2011 ▸; manufactured by Cinel Strumenti Scientifici S.r.l., Padova, Italy) is used to create an image of the X-ray source far downstream of the sample position. We refer to this beamline configuration as ‘collimated’. For microcrystal applications, the beam is typically focused with Kirkpatrick–Baez (KB) mirrors to 5 × 10 µm (vertical × horizontal) with a typical total flux of 1–2 × 10^13^ photons s^−1^. Toggling between the ‘collimated’ and the ‘microfocus’ configurations is accomplished automatically in less than 30 s by moving the KB mirrors into or out of the X-ray beam and readjusting the diffractometer and detector positions. The ‘unfocused’ configuration of the beamline refers to a configuration in which neither compound refractive lenses nor X-ray mirrors interfere with the beam. The beamline is controlled via the *MXCuBE* graphical user interface (Gabadinho *et al.*, 2010 ▸; Oscarsson *et al.*, 2019 ▸); experimental parameters and intermediate results are stored in the attached ISPyB database (Delagenière *et al.*, 2011 ▸).

The sample stage at P14 is an MD3 diffractometer (Arinax, Moirans, France; Cipriani *et al.*, 2007 ▸) equipped with a high-precision kappa diffractometer. Sample rotation is realized with a <100 nm sphere of confusion for the vertical and downward Ω axis combined with the sample-centering stage. An on-axis viewing system consisting of a high-resolution zoomable optical microscope (Supplementary Fig. S1) is integrated into the diffractometer and allows accurate interactive centering of samples with respect to the X-ray beam.

The detector-translation stage as designed in-house and for standard crystallographic beamline operation carries an EIGER 16M detector (DECTRIS, Baden, Switzerland). The stage offers five degrees of freedom (vertical and horizontal translation, roll, pitch and yaw). The sample-to-detector distance is adjustable between 10 cm and 3 m.

### 2.3. Diffraction rastering   

For diffraction raster scanning we used the beamline in microfocus configuration at a photon energy of 12.7 keV with a flux of 1.2 × 10^13^ photons s^−1^ through a beam cross-section of 5 × 10 µm (FWHM of an approximately Gaussian beam profile). Rastering was performed via a series of shutterless parallel helical scans (Gati *et al.*, 2014 ▸). The diffracted X-rays were recorded using a 4M region of interest of the EIGER 16M detector located at a distance of 289 mm from the sample position. The chosen combination of X-ray energy, detector size and crystal-to-detector distance limited the maximally reachable resolution at the detector edge to 2.0 Å. During a raster scan, the strength of the diffraction signal was evaluated on the fly using *Dozor* (Popov & Bourenkov, 2016 ▸) as implemented in the *EDNA* online data-analysis system (Incardona *et al.*, 2009 ▸), displayed as a heat plot in the *MXCuBE* beamline interface and stored in ISPyB. The X-ray dose deposited in the sample during the scan was estimated with *RADDOSE* (Paithankar *et al.*, 2009 ▸).

### 2.4. X-ray imaging   

For X-ray imaging experiments, we used the unfocused configuration of the beamline at an X-ray energy of 12.7 keV. Samples were mounted on the diffractometer and images were recorded using an X-ray imaging system consisting of a thin (2.6 µm) GGG:Eu scintillator (CEA-Leti, Grenoble, France), a 45° mirror reflecting the image of the scintillator upwards, an Olympus UPlanFI 20-fold objective (Olympus, Tokyo, Japan) and a Dalsa Pantera TF 1M60 CCD camera (Teledyne, Waterloo, Canada) with 1024 × 1024 pixels and 1–58 fps acquisition frequency. Taking into account all optical elements, the imaging setup resulted in an effective pixel size of 0.6 µm, covering a field of view of 614 × 614 µm. For tomographic data collection, the goniometer motor was set to rotate at a constant velocity under closed-loop control with an error of <0.001°, while frame acquisition was timed independently by the internal camera clock with a precision of better than 1 µs. Thus, the relative rotation angles between frames were well defined. Starting and stopping of the camera were not synchronized with the rotation axis, *i.e.* absolute rotation angles were not registered. The fast X-ray shutter was synchronized with the goniometer rotation axis and remained open during data acquisition. In the following, we denote the distance between the sample and the scintillator as the sample-to-camera distance.

### 2.5. X-ray microscopy   

To magnify the X-ray image detected by the scintillator, we introduced a compound refractive lens (CRL) consisting of an adjustable number of parabolic beryllium X-ray refractive lenses with radii of 50 µm (RXOPTICS, Aachen, Germany; Lengeler *et al.*, 2001 ▸) mounted in a small housing (RX­OPTICS, Aachen, Germany) as an objective between the sample and the X-ray camera [Fig. 1 ▸(*c*)]. In this configuration, the imaging camera was fixed on an *xy* stage on the hutch wall 5 m downstream of the sample. The CRL objective was installed on the detector stage [Fig. 1 ▸(*b*)] and remotely aligned with the available degrees of freedom of the detector stage. By optimization of the X-ray energy, the sample-to-objective distance and the number of refractive lenses in the objective, up to 25-fold magnification of the image generated on the scintillator can in principle be achieved.

To record images in the microscopic setting, we increased the flux density in the field of view by approximately a factor of 50 by increasing the number of lenses in the beam in the white-beam transfocator.

### 2.6. X-ray spectra   

It should be noted that for both the unfocused (without CRL) and the collimated (with CRL) configurations of the beamlines the influence of higher harmonics is negligible. Considering the undulator spectrum, monochromator reflectivity, beamline transmission and the efficiency of the scintillator, the contribution of the third harmonic to the recorded X-ray image is less than 0.5% in unfocused mode. In collimated mode, owing to the energy dependency of the refractive index, the CRL essentially only acts on the first and not on the third harmonic and thus further reduces the high-energy contribution 50-fold.

### 2.7. Image processing   

We corrected the images acquired with the Dalsa camera by a flat field to ensure maximum contrast and maximum signal to noise. For this correction, we firstly collected a set of 30 X-ray images without any sample, representing slightly different illumination conditions owing to fluctuations in the X-ray beam. Secondly, each image taken on a sample was corrected by dividing it by the flat field with the highest similarity, using the similarity index (SSIM) as implemented in the *scikit-image* Python module (van der Walt *et al.*, 2014 ▸) as a metric.

For tomographic reconstruction of the sample, we performed phase retrieval from the flat-field-corrected images using the *ANKAphase* 2.1 software (Weitkamp *et al.*, 2011 ▸), based on the single-distance non-iterative phase-retrieval algorithm as described in Paganin *et al.* (2002 ▸), employing an empirically optimized δ/β ratio of 5 × 10^3^. The resulting images were subjected to a Fourier wavelet filter (Münch *et al.*, 2009 ▸) to remove vertical stripes from the sinogram, reducing ring artifacts and minimizing phase-contrast aliasing in downstream processing steps. The actual tomographic reconstruction was performed with the *tomopy* 1.1.2 Python package (Gürsoy *et al.*, 2014 ▸) using the built-in *Gridrec* algorithm and Shepp–Logan filter with default settings.

A typical sequence of flat-field correction, phase retrieval and tomographic reconstruction based on 180 raw projection images took ∼1.5 min on a four-CPU iMac with a 3.8 GHz Intel Core i5.

For segmentation of the 3D tomogram into regions containing crystals or the micromesh used for mounting and regions corresponding to lipidic cubic phase or air surrounding the sample, we employed the carving workflow in *Ilastik* 1.3.0 (Sommer *et al.*, 2011 ▸). After selecting 466 sequential tomographic slices comprising the regions of interest of the sample, we used the ‘step edges’ edge filter at a σ level of 2.5 to define boundaries. Subsequently, the seeded watershed algorithm was used iteratively to segment the tomogram. After automated segmentation based on manually defined ‘object’ and ‘background’ seeds, the predicted segmentation was refined iteratively by manually placing additional markers followed by automated segmentation until a clear and accurate separation between crystals and micromesh versus lipidic cubic phase and air was achieved. The result of the segmentation was exported as a 3D mesh into an .obj file.

## 3. Results and discussion   

### 3.1. Determination of source size and coherent fraction   

**Figure 2:**
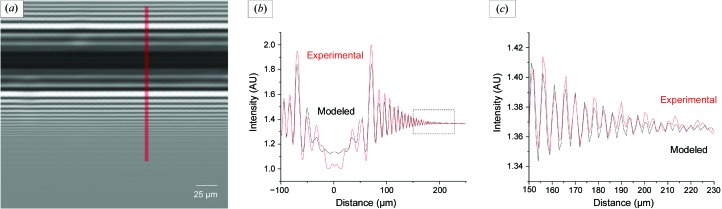
X-ray interference pattern from a boron fiber. (*a*) Intensity distribution acquired with the X-ray camera placed at a distance of 5 m from the point of intersection between the boron fiber and the 12.7 keV X-ray beam. (*b*) The cross-section of (*a*) (red) and the predicted intensity distribution for an effective source size of 35 µm (black). X-ray intensity (in arbitrary units) is measured as a function of distance from the core of the fiber. The experimental profile was obtained by averaging over ten pixel columns [red line in (*a*)]. (*c*) shows a magnification of the rectangular inset in (*b*).

To characterize the coherence properties of the X-ray beam of beamline P14, we exposed a horizontally mounted boron wire of 100 µm diameter with a 15 µm diameter tungsten core (Goodfellow Cambridge, order code 988-350-69) to the unfocused X-ray beam of P14 at an X-ray energy of 12.7 keV. The resulting interference pattern was recorded with the X-ray camera placed at distance of 5 m from the sample using an exposure time of 17 ms [Fig. 2 ▸(*a*)]. The flat-field-corrected image obtained was very clear and homogeneous, indicating the high quality of the X-ray beam and the flat-field correction.

Following the formalism to describe the above experiment in terms of an inline holography model as developed by Kohn *et al.* (2000 ▸), we analyzed the acquired image with the software described by Kohn *et al.* (2001 ▸). Briefly, using this software, by analysis of the number of detectable fringes and their visibility via a fit between the experimentally observed fringes and corresponding fringes simulated from an analytical description of the interference process [Figs. 2 ▸(*b*) and 2 ▸(*c*), Supplementary Fig. S2], the properties of the X-ray beam used can be derived. The best match between experimental and simulated intensity distributions was observed by assuming a vertical ‘effective source size’ of *d*_eff_ = 35 (±3) µm (FWHM of the intensity distribution). The difference between this estimated effective source size and the nominal vertical source size of 13 µm (FWHM) for an U29 undulator at a photon energy of 12 keV can be attributed to broadening of the X-ray beam caused by (i) thermal deformation of the surface of the monochromator crystals and (ii) high-frequency vibrations (>80 Hz) induced by the cryogenic cooling of the monochromator crystals. Measurements of the effective source size on other synchrotron beamlines using coherent scattering from a boron fiber have resulted in comparable values. For example, for ID22 at ESRF *d*_eff_ values were determined to be 35 (±4) µm (Kohn *et al.*, 2001 ▸), while a nominal size of 15 µm (Dimper *et al.*, 2015 ▸) was expected.

According to (1), an effective source size of 35 (±3) µm corresponds to a transversal coherence length of 170 (±13) µm at the sample position. For the reasons given above, this number is approximately three times smaller than the value of 500 µm (FWHM) for the vertical coherence length of PETRA III calculated for the theoretical vertical source size of the U29 undulator at 12.7 keV photon energy and at a distance of 61 m from the source. The transversal coherence length measured here compares well with the analogous value, reported as 277 µm at a distance of 91 m from the source at a photon energy of 8 keV, for beamline P10 at PETRA III (Zozulya *et al.*, 2012 ▸).

The increase in effective source size by the change in the X-ray directional distribution at the double-crystal monochromator can be estimated by a convolution of the theor­etical ray distribution at the source point with the angular spread owing to the monochromator. Assuming an angular spread of ΔΦ_DCM_ = 0.7 µrad owing to the monochromator situated at *L*_DCM_ = 45 m from the source point and projected back to the source point with a nominal source size of *d*_V_ = 13 µm and *d*_H_ = 330 µm, the effective source sizes for the vertical and horizontal directions, *d*_eff-V,H_ can be modeled as
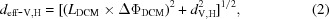
resulting in
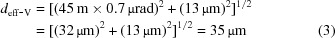
and, assuming identical broadening of the X-rays for both the vertical and the horizontal directions,
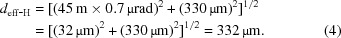



Thus, in contrast to the vertical direction, the effective source size (and thus the expected transversal coherence length) in the horizontal direction is only minimally affected by the broadening of the X-ray distribution at the monochromator and remains within the limit of 400 µm as derived above (1) for achieving 1 µm resolution.

In addition to the high degree of transversal spatial coherence measured, the excellent uniformity of the interference pattern [Fig. 2 ▸(*a*)] indicates high homogeneity of the wavefront at the sample position.

### 3.2. Optical visualization   

**Figure 3:**
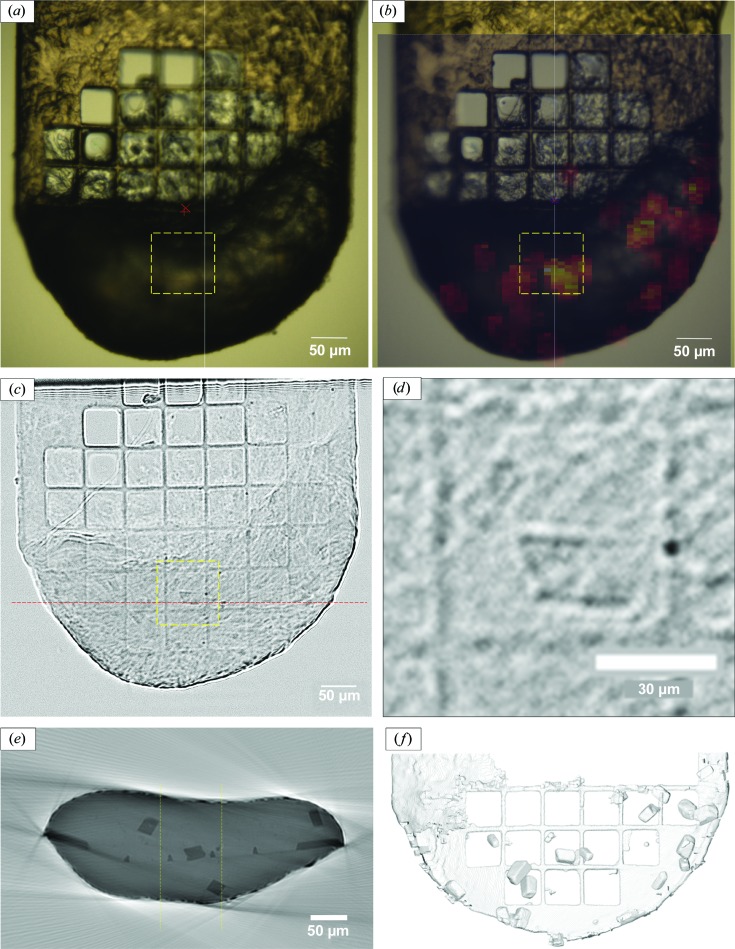
Visualization of crystals embedded in an LCP matrix. (*a*) Image taken with the on-axis microscope of the MD3 diffractometer. (*b*) Heat plot of the number of diffraction spots found by *Dozor* as a function of *x*–*y* positions tested with a microfocus beam. Pseudo-colors represent the number of diffraction spots per image on a linear scale using the ‘autumn’ colormap (https://matplotlib.org). The highest number of 1300 spots (indicated by a white coloring for the corresponding *x*–*y* position) was found for a crystal diffracting to a resolution of 2.0 Å. (*c*) A flat-field-corrected projection recorded by X-ray imaging. (*d*) Enlargement of the region marked in (*a*)–(*c*). (*e*) Ortho-slice through the 3D tomogram derived from 180 X-ray projection images taken at the *y* coordinate indicated by the dashed red line in (*c*). The grayscaling is proportional to the attenuation coefficient. (*f*) 3D image after identification of regions representing crystals or the mesh mount using iterative segmentation as implemented in the carving workflow of *Ilastik*. The figure was produced using *GLC_Player* (http://www.glc-player.net/).

Crystals of lysozyme as grown in LCP were transferred to a kapton micromesh (MiTeGen, USA) attached to a standard crystallographic SPINE pin. The pin was mounted onto the diffractometer into a cryostream at 100 K. As expected (Cherezov *et al.*, 2009 ▸), the lipidic cubic phase became opaque to visible light upon cryogenic cooling, so that crystals were not detectable with the on-axis optical microscope as integrated with the MD3 diffractometer [Fig. 3 ▸(*a*)].

### 3.3. Diffraction rastering   

To localize crystals in the opaque lipidic cubic phase, we performed a diffraction raster scan with the sample holder perpendicular to the beam covering an area of 500 × 500 µm with a vertical sample displacement of 5 µm between frames and a horizontal displacement of 10 µm between parallel vertical lines, resulting in the collection of a total of 4998 frames. With an exposure time of 7.5 ms per frame, the total time to complete the raster scan was 73 s. During the raster scan ∼560 kGy, corresponding to ∼2% of the proposed dose limit of 30 MGy (Owen *et al.*, 2006 ▸), was deposited in the irradiated part of the sample. As determined by on-the-fly data analysis, 780 of the collected frames contained more than 20 diffraction spots. In the corresponding heat plot [Fig. 3 ▸(*b*)], approximately 20 regions containing crystalline material could be detected. In the chosen projection, the crystals appear to be homogeneous in size but with varying diffraction power, where the latter may be attributed to orientation-dependent diffraction power.

### 3.4. Phase-contrast imaging   

For phase-contrast imaging of the cooled LCP sample mounted on a kapton micromesh, we illuminated the sample with the unfocused beam at an energy of 12.7 keV with a sample-to-camera distance of 110 mm. A single 17 ms exposure in a face-on orientation of the micromesh clearly revealed the outlines of the crystals contained in the LCP matrix [Fig. 3 ▸(*c*)]. Close inspection of the image shows that the achieved resolution is close to the pixel size of the CCD camera of 0.6 µm. The X-ray dose deposited in the sample for recording one projection image with the unfocused beam is of the order of 80 Gy and therefore is ∼7000 times smaller than the dose applied for one 2D diffraction scan with a microfocus beam.

To obtain a three-dimensional view, we recorded 180 projection images with 17 ms exposure each and steps of 1° rotation between individual exposures in a total time of 3 s. When stacking the projections and displaying them continuously, a clear view of where the crystals are located in 3D can be obtained (see the video in the supporting information).

From the set of 180 projection images, a 3D tomogram was assembled using standard methods. In the 3D tomogram, the contrast between crystals and the LCP matrix is markedly enhanced [see Fig. 3 ▸(*e*)] and the crystals can be clearly located along all three dimensions. The total dose deposited in the sample to collect all data necessary for a full 3D reconstruction of the sample was estimated to be of the order of 15 kGy, *i.e.* less than 0.1% of the dose expected to be tolerated by a typical macromolecular crystal.

Analysis of the 3D reconstruction clearly revealed the localization and shapes of the crystals present in the LCP matrix [Fig. 3 ▸(*f*)].

### 3.5. X-ray microscopy with refractive lenses   

Using a low-divergence X-ray beam as available on P14 and for realistic sample-to-camera distances, the resolution of the X-ray imaging setup is limited by the effective pixel size of the X-ray camera. By placing a CRL as an objective downstream of the sample, the X-ray image produced by the sample can be magnified (Lengeler *et al.*, 2003 ▸) before interacting with the scintillator.

For imaging details of our samples, we therefore placed a CRL consisting of 20 individual refractive beryllium lenses onto the P14 detector stage. To further increase the magnification factor, the X-ray energy used for imaging was reduced from 12.7 to 10 keV. The effective aperture *A*_eff_ (Kohn *et al.*, 2003 ▸; Kohn, 2017 ▸) and the focal distance *F* of the CRL at 10 keV were estimated to be *A*_eff_ = 270 µm and *F* = 37 cm, respectively.

Following the thin-lens equation
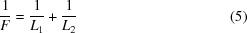
we positioned the objective at a distance *L*_1_ = 40 cm from the sample and at a distance *L*_2_ = 456 cm from the X-ray camera, thus reaching a nominal magnification of *L*_2_/*L*_1_ = 11.4, giving an effective camera pixel size of 52 nm, which is somewhat smaller than the optical resolution of the objective lens as limited by the diffraction limit δ, which can be calculated as
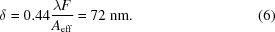
Owing to the small effective camera pixel size and a reduction in X-ray intensity owing to absorption in the objective CRL, we switched the beamline into collimated mode to increase the X-ray illumination of the sample. Using the white-beam transfocator in a configuration with two refractive lenses of apical radii *R* of 2000 and 1000 µm, respectively, plus five lenses with *R* = 500 µm, forming one seven-lens CRL, as a condenser, we increased the X-ray flux density by 50 times (up to 1.5 × 10^12^ photons s^−1^ into a 54 × 54 µm cross-section) with respect to the unfocused beamline configuration.

**Figure 4:**
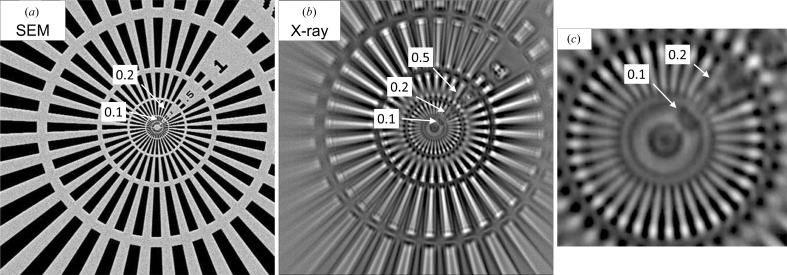
Scanning electron (*a*) and X-ray micrographs (*b*, *c*) of the Siemens star (Ta on SiN; XRESO-50HC, NTT-AT, Japan). Numbers along the upper right diagonal indicate feature sizes in µm. (*c*) Enlargement of the central part of (*b*) revealing the smallest distinguishable bars of sizes 0.1–0.2 µm.

Exposing a Siemens star to the collimated radiation, features at 0.1 µm were clearly discernible, indicating that the resolution of the complete imaging setup was of the order of 150 nm (Fig. 4 ▸), corresponding to three pixels on the CCD detector. The image of the Siemens star also displays high uniformity and indicates the absence of spherical lens aberrations and parasitic perturbations in the wavefront reaching the sample.

**Figure 5:**
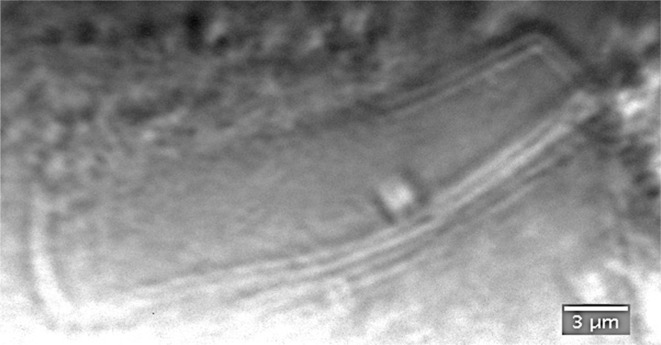
Flat-field-corrected X-ray micrograph of a protein crystal embedded in LCP magnified by a factor of 11.4 by an objective CRL placed between the sample and the X-ray camera.

We then recorded a micrograph of a crystal localized in the mount used for the previous tomographic experiments. As seen from the high-resolution image (Fig. 5 ▸), phase-contrast X-ray microscopy visualizes deformed crystal boundaries. Based on the uniformity in the image of the Siemens star taken under the same experimental conditions, it can be excluded that this deformation is an imaging artifact. Here, it should be noted that owing to the higher photon flux density and the longer exposure time used, the dose deposited in the sample for recording a single projection is more than 4000 times higher than for the recording of a projection image under imaging conditions (Table 1 ▸).

## 4. Conclusions and perspectives   

With advances in diffraction data-collection technologies, ever more challenging macromolecular systems have become amenable to crystallo­graphic structure determination. Many of these systems will give rise to only small crystals that additionally may be embedded in matrices that are highly refractive or opaque to optical light. Here, we have demonstrated that X-rays as available on a macromolecular crystallography beamline can be used to visualize crystals that are otherwise difficult to detect, with X-ray dose and image-acquisition times that are compatible with macromolecular crystallography experiments.

Based on an X-ray interference experiment on a boron fiber, we have measured the effective vertical source size of beamline P14 to be of the order of 35 (±3) µm (FWHM). At a distance of 61 m from the source point, this source size corresponds to a transversal coherence length of 170 (±13) µm (FWHM). Both parameters were determined with a double-crystal monochromator present in the beam and can probably be improved by minimizing the wavefront distortions caused by surface inhomogeneities and/or vibrations of the monochromator crystals present in the beamline.

The most widely used method for localizing macromolecular crystals in opaque matrices is based on a raster scan of the sample with a microfocus beam. For a typical field of view such as that selected here (500 × 500 µm), this procedure is time-consuming (on the minute scale), uses a significant fraction of the dose generally tolerated by a cryocooled macromolecular crystal to localize it instead to collect diffraction data, and results in a diffraction heat map with a resolution limited by the dimensions of the microfocus beam used, here of the order of 5–10 µm. In contrast, full-field phase-contrast imaging allows the imaging of a region of interest encompassing the entire sample (here, 614 × 614 µm) on a millisecond time scale with an X-ray dose lower by a factor of more than 5000 in comparison to the raster scan, resulting in an image with resolution in the single-micrometre range, allowing the clear visualization of crystals with linear dimensions down to the micrometre range.

The limited requirements in terms of dose and wall-clock time to acquire a full-field phase-contrast image allow a full tomographic series of images (*e.g.* 180 images spaced by 1° rotations) to be acquired on a time scale of seconds with a total X-ray dose that is still negligible compared with the total dose tolerated by a typical macromolecular crystal. Three-dimensional tomograms can be derived in less than 2 min and clearly show boundaries of the crystals in three dimensions.

By adding a compound refractive lens into the optical path between the sample and the scintillator, we have shown that the effective resolution of the setup can be significantly increased. However, for practical application of this microscopy mode, there are several caveats. Firstly, to record higher resolution images, the deposited X-ray dose has to be increased substantially (Du & Jacobsen, 2018 ▸). Secondly, owing to the decreased field of view (54 × 54 µm), standard 3D tomographic data can only be obtained from small samples that fit completely into the available field of view. Larger samples will require specific tomographic approaches for dealing with the truncated parts of projections at the expense of decreased image quality (Kyrieleis *et al.*, 2011 ▸) or elaborate data-collection strategies, resulting in increased experimental times and X-ray doses (Haberthür *et al.*, 2010 ▸). Thirdly, the CRL as introduced downstream of the sample position acts as a phase object and at present makes full phase retrieval practically impossible (Kohn, 2003 ▸). Nevertheless, our experiment has shown that recording 2D projection images in a microscopy mode, possibly applying a rastering strategy, could still allow the identification of the shapes and positions of crystals, especially when mounted in thin films, as is often the case for crystals mounted in loop-shaped holders.

The imaging experiments presented here were performed on a standard protein crystallography beamline. Use of the existing optical microscopy for pre-alignment, the diffracto­meter for highly precise positioning and for sample rotation to acquire tomographic series, and the existing motorization of the detector table for rapid toggling between the collection of X-ray diffraction or X-ray imaging data allows swift integration of X-ray imaging of crystal mounts into the standard workflow of crystallographic data collection. As a first step, we are pursuing a project towards presenting the user with a 3D tomogram for three-click centering in the *MXCuBE* user interface (Oscarsson *et al.*, 2019 ▸) in operation on P14. At a later stage, a 3D tomogram could be automatically acquired and crystals sought using available segmentation algorithms (Spina *et al.*, 2018 ▸). The localization of crystals in X-ray-based projections or tomograms also effectively removes the problem of the inaccurate location of crystals with visible light owing to refraction at the air–mounting matrix interface (Bowler *et al.*, 2016 ▸). Given the simplicity of the imaging setup and its compatibility with diffraction instrumentation, similar procedures could be implemented on many other macromolecular crystallography beamlines.

The successful semi-automatic segmentation of the tomogram revealing the sample holder and the crystals holds the potential to obtain a segmented reconstruction of the entire sample, including the embedding material. Knowledge of the shape and orientation of the crystals could be used to dynamically adjust the slit settings to follow the projection of the crystal during a rotation data collection in order to reduce the background. Information from segmentation could also be used to derive parameters for analytical absorption corrections. Such analytical corrections could be a more accurate replacement for the currently used empirical absorption corrections, allowing the use of even weaker anomalous signals for crystallographic phasing. As pointed out by Brock­hauser *et al.* (2008 ▸), high-resolution 3D imaging data for the purpose of deriving absorption corrections could actually be collected after the diffraction data collection so as not to compromise highly accurate anomalous diffraction data by radiation damage.

Given the high quality of the images obtained from a boron fiber and protein crystals, we are currently evaluating whether the coherence and the wavefront homogeneity available on P14 can be exploited to image other biological samples such as cells, tissues or insects in full-field mode without the limitations in imaging penetration depth as in electron microscopy. Using the available robotic sample-mounting systems, a high-throughput full-field high-energy phase-contrast imaging instrument could possibly be realized with a relatively small effort.

While the current generation of synchrotrons has enabled highly interesting applications of X-ray imaging technologies, further improvements in beam properties in the next generation of low-emittance, possibly diffraction-limited synchrotron light sources such as NSLS II (Wang *et al.*, 2016 ▸), MAX IV (Tavares *et al.*, 2014 ▸), ESRF-EBS (Dimper *et al.*, 2015 ▸) and PETRA IV (Schroer *et al.*, 2018 ▸) will pave the way to more robust imaging methodologies delivering more accurate images at higher resolution.

## Supplementary Material

Supplementary Figures. DOI: 10.1107/S2059798319011379/gm5064sup1.pdf

X-ray projection images taken during rotation of the sample in avi format. DOI: 10.1107/S2059798319011379/gm5064sup2.avi

Segmented 3D tomogram in obj format. DOI: 10.1107/S2059798319011379/gm5064sup3.txt

## Figures and Tables

**Table 1:** Experimental parameters and estimated X-ray doses for raster scanning and different imaging procedures

	Raster scan	Imaging (single shot)	Tomography (180 projections)	X-ray microscopy (single shot)
Flux (photons s^−1^)	1.2 × 10^13^	4 × 10^12^	4 × 10^12^	1.5 × 10^12^
Beam size (µm)	5 × 10	614 × 614	614 × 614	54 × 54
Flux density (photons s^−1^ mm^−2^)	2.4 × 10^17^	1 × 10^13^	1 × 10^13^	5.1 × 10^14^
Resolution (µm)	5 × 10	0.6 × 0.6	0.6 × 0.6	0.15 × 0.15
Total exposure time (s)	37.5	0.017	3	1
Total collection time (s)	73	0.017	3	1
Dose (kGy)	560	0.076	15	330
