# Supplementary material for: Visualization of protein crystals by high-energy phase-contrast X-ray imaging
Source: Acta Crystallogr D Struct Biol. 2019 Oct 31;75(Pt 11):947–58. doi: 10.1107/S2059798319011379 (PMC6834075; doi:10.1107/S2059798319011379)
Supplement: Supplementary file 1 [file d-75-00947-sup1.pdf]

STRUCTURAL
BIOLOGY

Volume 75 (2019)

Supporting information for article:

Visualization of protein crystals by high-energy phase-contrast X-ray imaging

Maxim Polikarpov, Gleb Bourenkov, Irina Snigireva, Anatoly Snigirev, Sophie Zimmerman, Krisztian Csanko, Sandor Brockhauser and Thomas Schneider

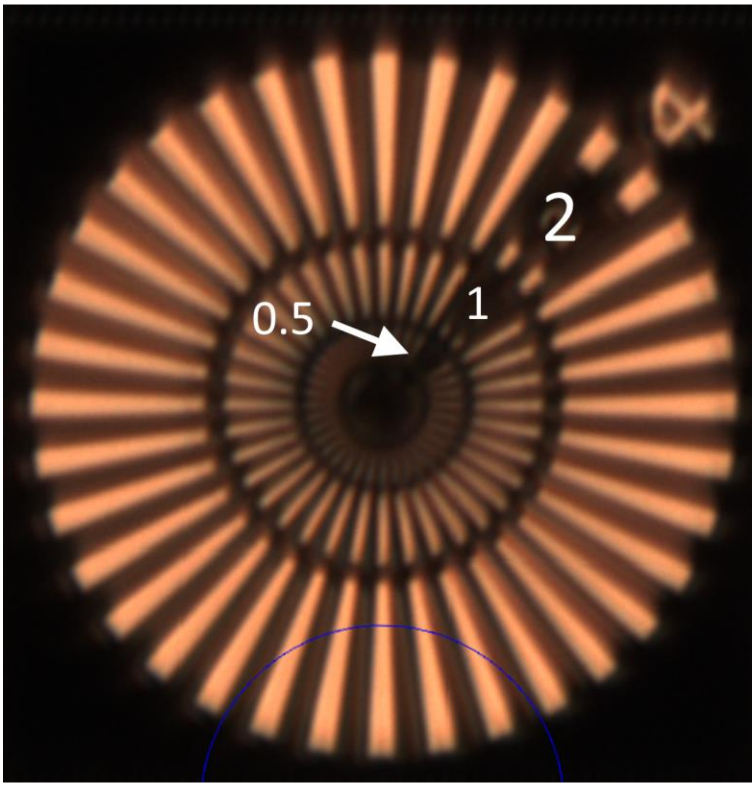

Figure S1 Optical micrograph of the Siemens star taken with the on-axis viewing system on the MD3 diffractometer. Bars with the size of 500 nm are the last visible, thus defining the optical resolution.

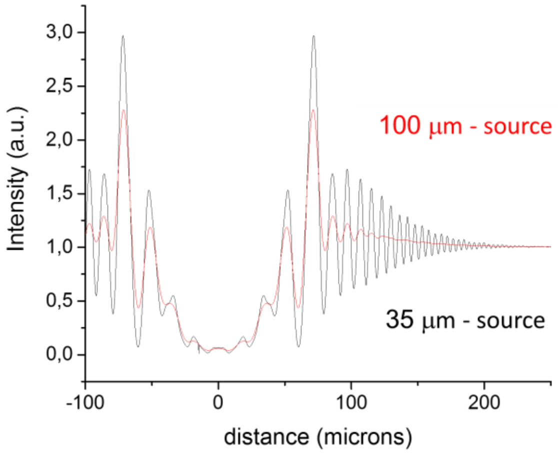

Figure S2 Predicted intensity distributions as a function of distance for effective source sizes of 35 µm (in black) and 100 µm (in red) illustrating the influence of the assumed source size on the visibility of fringes. Calculations are made for the experimental conditions described in paragraph 3.1.
